# Enhanced Chemical Stability of Tetramethylammonium Head Groups via Deep Eutectic Solvent: A Computational Study

**DOI:** 10.3390/molecules29204869

**Published:** 2024-10-14

**Authors:** Mirat Karibayev, Bauyrzhan Myrzakhmetov, Yanwei Wang, Almagul Mentbayeva

**Affiliations:** 1 Department of Chemical & Materials Engineering, School of Engineering and Digital Sciences, Nazarbayev University, Astana 010000, Kazakhstan; 2 Center for Energy and Advanced Materials Science, National Laboratory Astana, Nazarbayev University, Astana 010000, Kazakhstan

**Keywords:** tetramethylammonium head groups, deep eutectic solvent, anion exchange membranes, chemical stability, density functional theory, molecular dynamics, ylide formation, nucleophilic substitution

## Abstract

The chemical stability of tetramethylammonium (TMA) head groups, both with and without the presence of a choline chloride and ethylene glycol-based deep eutectic solvent (DES), was studied using Density Functional Theory (DFT) calculations and *ab initio* Molecular Dynamics (MD) simulations. DFT calculations of transition state energetics ($\Delta E_{\mathrm{reaction}}$, $\Delta G_{\mathrm{reaction}}$, $\Delta E_{\mathrm{activation}}$, and $\Delta G_{\mathrm{activation}}$) for key degradation mechanisms, ylide formation (YF) and nucleophilic substitution ($S_{N}2$), suggested that the presence of DES enhances the stability of the TMA head groups compared to systems without DES. *Ab initio* MD simulations across hydration levels (HLs) 1 to 5 indicated that without DES, YF dominates at lower HLs, while $S_{N}2$ does not occur. In contrast, both mechanisms are suppressed in the presence of DES. Temperature also plays a role: without DES, YF dominates at 298 K, while $S_{N}2$ becomes prominent at 320 K and 350 K. With DES, both degradation mechanisms are inhibited. These findings suggest DES could improve the chemical stability of TMA head groups in anion exchange membranes.

## 1. Introduction

Anion exchange membrane (AEM) fuel cells have gained significant attention due to their cost-effectiveness and high energy conversion efficiency [1,2,3,4,5,6,7,8]. In AEMs, quaternary ammonium (QA) head groups covalently bound to the polymer backbone are susceptible to chemical degradation under alkaline conditions and at elevated temperatures [9,10,11,12]. Improving the chemical stability of these QA head groups is critical for enhancing the long-term performance of AEMs in fuel cell applications.

In our recent computational studies, we investigated how the chemical structure of QA head groups affects their stability under alkaline conditions. For example, in one of our works, we explored the degradation mechanisms of trimethylhexylammonium (TMHA) and benzyltrimethylammonium (BTMA) head groups, focusing on key degradation pathways such as nucleophilic substitution ($S_{N}2$) and ylide formation (YF) [13]. In another study, we examined the influence of hydration levels (HLs) on the chemical stability and transport properties of quaternized chitosan (QCS) head groups in AEMs, revealing how water content affects degradation under alkaline conditions [14]. These investigations, along with other studies in the literature, highlight the importance of both structural and environmental factors in determining the stability of QA head groups. Chempath et al. demonstrated that water content and solvation play a critical role in the stability of QA cations, with nucleophilic substitution and ylide formation being key degradation pathways in alkaline environments [15,16]. Similarly, Dekel et al. emphasized the importance of HLs, showing that reduced water content accelerates degradation through nucleophilic attack by hydroxide ions [17].

To enhance the stability of QA groups, structural modifications such as the use of aromatic cations like pyridinium have been explored, as they are less prone to Hofmann elimination, though they remain susceptible to nucleophilic substitution if not adequately protected [18]. Recent studies have also focused on alternative cations such as piperidinium and quinuclidinium, which exhibit superior alkaline stability compared to traditional QA groups. For example, Zhao et al. demonstrated that bis-piperidinium-based AEMs exhibit excellent stability due to the absence of ether linkages and the inclusion of flexible alkyl spacers [19], while Zeng et al. showed that N-methylquinuclidinium-based AEMs maintain ultra-high stability in concentrated alkaline solutions [20]. Moreover, the incorporation of these modifications, along with crosslinking strategies and ether-bond-free polymer backbones, has significantly improved the overall chemical stability of AEMs [18,21,22].

Building on this prior work, we now shift our focus from the chemical structure of QA head groups to the effect of the solution environment, specifically the impact of Deep Eutectic Solvent (DES). DES, which is a eutectic mixture of Lewis or Brønsted acids and bases, exhibits several favorable properties, including biodegradability, non-toxicity, and low vapor pressure [23,24,25,26,27,28,29]. Recent studies have shown that integrating DES into polymer electrolytes can significantly enhance both performance and stability, particularly in lithium-metal batteries, where they improve safety and overall efficiency [30,31,32]. Additionally, DES has been explored as potential electrolytes in proton exchange membrane fuel cells (PEMFCs), achieving higher power densities and improved thermal stability [33]. Beyond energy storage, DES has found applications in membrane fabrication and separation processes, driven by their environmentally friendly properties [34]. Notably, DES-supported polymer-based AEMs, such as those developed using poly(vinyl alcohol) nanofibers, have demonstrated enhanced ionic conductivity and mechanical stability compared to traditional AEMs, effectively addressing challenges like low stability in alkaline environments [35]. These advancements highlight the growing importance of DES in fuel cell technologies and other membrane-based applications. However, despite its potential to improve the stability of chemical systems, the role of DES in stabilizing QA head groups in AEMs remains underexplored.

In this work, we employed Density Functional Theory (DFT) calculations and *ab initio* Molecular Dynamics (MD) simulations to study the chemical stability of tetramethylammonium (TMA) head groups, both with and without the presence of a choline chloride and ethylene glycol-based DES. The interactions between hydroxide (${\mathrm{OH}}^{-}$) ions and TMA head groups were analyzed across various HLs and temperatures. By incorporating DES molecules, we aimed to explore their role in modulating the degradation mechanisms of TMA head groups, providing insights into the development of more chemically robust AEM materials.

In the following sections, we present our computational approach, including the DFT and *ab initio* MD methods, followed by an in-depth analysis of the stability of TMA head groups under different HLs and temperatures. This study offers new insights into the suppression of degradation mechanisms through the addition of DES, contributing to advancements in AEM-based renewable energy technologies.

## 2. Model and Methods

### 2.1. System of Interest

**Figure 1:**
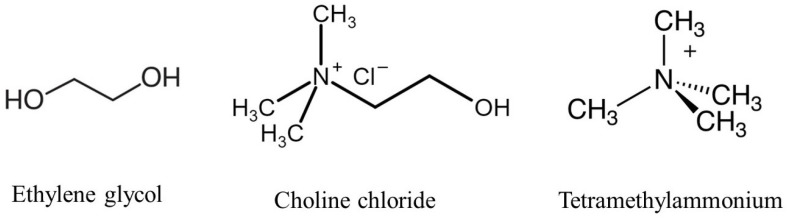
Molecular structures of the key components in the study, including choline chloride–ethylene glycol-based DES and TMA head group.

In this study, we modeled a choline chloride and ethylene glycol-based DES, as shown in Figure 1, at a 1:2 molar ratio. The DES was combined with QA head groups, ${\mathrm{OH}}^{-}$ ions, and water molecules to simulate the environment relevant to AEMs. These components were used to assess the chemical stability of TMA head groups under various conditions. The TMA head group of the AEM segment was chosen as the model system, and its interactions with ${\mathrm{OH}}^{-}$ ions were studied in both the presence and absence of DES. This computational model was used for DFT calculations and *ab initio* MD simulations to evaluate how DES influences the degradation mechanisms of the head groups, with a particular focus on varying HLs and temperatures. The aim was to simulate the behavior of the AEM in DES-supported environments and provide insight into how these factors contribute to the chemical stability of the TMA head groups.

### 2.2. DFT Calculations

DFT calculations were employed to optimize the electronic ground state geometries and perform frequency calculations, yielding key thermodynamic parameters: reaction energy ($\Delta E_{\mathrm{reaction}}$), Gibbs free energy change ($\Delta G_{\mathrm{reaction}}$), activation energy ($\Delta E_{\mathrm{activation}}$), and activation Gibbs free energy ($\Delta G_{\mathrm{activation}}$). These calculations were crucial for understanding the stability of TMA head groups in AEMs and the degradation mechanisms triggered by interactions with ${\mathrm{OH}}^{-}$. The hydroxide ions were assumed to originate from KOH or NaOH solutions used experimentally, reflecting the conditions typically present in AEM environments. The B3LYP functional, with the 6-311++g(2d,p) basis set, was used in conjunction with the polarizable continuum model (PCM) to simulate solvent effects [36,37]. The choice of the B3LYP functional is supported by its proven performance in calculating accurate thermodynamic properties and geometries, as demonstrated by Gill et al. [38]. Additionally, the use of the 6-311++g(2d,p) basis set follows the recommendations for highly accurate Gaussian basis sets provided by de Castro and Jorge [39]. The optimization of TMA head groups was carried out in the presence and absence of ${\mathrm{OH}}^{-}$ ions, both with and without DES.

To model the $S_{N}2$ and YF degradation mechanisms, transition state structures were identified using the same level of theory, with DFT optimizations performed in implicit DMSO [17,40]. DMSO was selected due to its ability to represent a polar, aprotic environment, which is particularly suitable for stabilizing the transition state of the degradation mechanisms considered in this study. Previous studies have demonstrated that DMSO provides an effective environment for evaluating the stability of QA groups under low hydration conditions, allowing for detailed exploration of their behavior [17,40]. The hydration level was varied by explicitly adding water molecules to the system, and transition states were optimized to investigate the stability of the head groups under different hydration conditions. The calculated reaction and activation energies for both $S_{N}2$ and YF mechanisms were used to assess the stability of TMA head groups in the presence and absence of DES.

The chemical degradation mechanisms are represented by the following reactions: $S_{N}2$ and YF for both DES-supported and unsupported systems, as shown in Equations (1)–(6). $\Delta E_{\mathrm{reaction}}$ and $\Delta E_{\mathrm{activation}}$ were calculated using Equations (7) and (8), while $\Delta G_{\mathrm{reaction}}$ and $\Delta G_{\mathrm{activation}}$ were calculated using Equations (9) and (10). Additionally, basis set superposition error (BSSE) was corrected using the counterpoise method to ensure accurate transition state energy evaluations.

(1) $$N^{+}{\left({\mathrm{CH}}_{3}\right)}_{4}+{\mathrm{OH}}^{-}\to {\mathrm{CH}}_{3}\mathrm{OH}+N{\left({\mathrm{CH}}_{3}\right)}_{3}$$

(2) $$N^{+}{\left({\mathrm{CH}}_{3}\right)}_{4}+{\mathrm{OH}}^{-}\to H_{2}O+N{\left({\mathrm{CH}}_{3}\right)}_{3}\left({\mathrm{CH}}_{2}\right)$$

(3) $$H_{2}O+N{\left({\mathrm{CH}}_{3}\right)}_{3}\left({\mathrm{CH}}_{2}\right)\to {\mathrm{CH}}_{3}\mathrm{OH}+N{\left({\mathrm{CH}}_{3}\right)}_{3}$$

(4) $$\mathrm{DES}\left(N^{+}{\left({\mathrm{CH}}_{3}\right)}_{4}\right)+{\mathrm{OH}}^{-}\to {\mathrm{CH}}_{3}\mathrm{OH}+\mathrm{DES}\left(N{\left({\mathrm{CH}}_{3}\right)}_{3}\right)$$

(5) $$\mathrm{DES}\left(N^{+}{\left({\mathrm{CH}}_{3}\right)}_{4}\right)+{\mathrm{OH}}^{-}\to H_{2}O+\mathrm{DES}\left(N{\left({\mathrm{CH}}_{3}\right)}_{3}\left({\mathrm{CH}}_{2}\right)\right)$$

(6) $$H_{2}O+\mathrm{DES}\left(N{\left({\mathrm{CH}}_{3}\right)}_{3}\left({\mathrm{CH}}_{2}\right)\right)\to {\mathrm{CH}}_{3}\mathrm{OH}+\mathrm{DES}\left(N{\left({\mathrm{CH}}_{3}\right)}_{3}\right)$$

(7) $$\Delta E_{\mathrm{reaction}}=\sum E_{\mathrm{products}}-\sum E_{\mathrm{reactants}}$$

(8) $$\Delta E_{\mathrm{activation}}=E_{\mathrm{transition}\;\mathrm{state}}-\sum E_{\mathrm{reactants}}-E_{\mathrm{BSSE}}$$

(9) $$\Delta G_{\mathrm{reaction}}=\sum G_{\mathrm{products}}-\sum G_{\mathrm{reactants}}$$

(10) $$\Delta G_{\mathrm{activation}}=G_{\mathrm{transition}\;\mathrm{state}}-\sum G_{\mathrm{reactants}}$$

The DFT calculations for static electronic structure optimizations and frequency analyses were performed using the GAUSSIAN16 software package (Gaussian, Inc., Wallingford, CT, USA) [41]. Post-analysis was conducted using GaussView (v6.0) [42].

### 2.3. Ab Initio Molecular Dynamics

Each *ab initio* MD simulation began with an initial configuration consisting of the TMA head group, ${\mathrm{OH}}^{-}$ ion, and water molecules, placed in a simulation box with dimensions 1.50nm×1.50nm×1.50nm. Water molecules were added explicitly to tune the HL of the system, as the hydration state is critical to understanding the degradation mechanisms of AEMs in practical applications.

Atomic forces were computed using DFT with the BLYP functional. The Kohn–Sham orbitals were represented using a hybrid Gaussian plane-wave (GPW) approach, which combines Gaussian-type orbitals with plane-wave basis sets to efficiently construct the Kohn–Sham matrix while maintaining accuracy. Goedecker–Teter–Hutter (GTH) pseudopotentials were used to represent core electrons, with adjustments made for the density functionals employed. To optimize computational efficiency, matrix elements smaller than $10^{-10}$ were disregarded, and a plane-wave cutoff of 400 Ry was applied. The choice of this cutoff value was guided by a recent study by Alizadeh et al. [43], where a similar plane-wave cutoff was employed in DFT simulations of DES and demonstrated to provide a good balance between accuracy and computational efficiency. Dispersion forces were included using the Grimme D3 approximation, with a convergence criterion for the self-consistent field (SCF) set to $10^{-6}$.

Energy minimization was first performed under the NVE ensemble for 10 ps to optimize the initial configuration. Following this, the simulations were conducted for 50 ps using the NVT ensemble at reference temperatures of 298 K, 320 K, and 350 K. Temperature control was managed using the Generalized Langevin Equation (GLE) method [44], and periodic boundary conditions were applied in all directions [45]. All simulations were performed using the CP2K software suite (version 9.1) [46], and bond distances were analyzed at various HLs and temperatures using Visualization Molecular Dynamics (VMD) software (version 1.9.1) [47].

## 3. Results and Discussion

### 3.1. Electrostatic Potential Map

**Figure 2:**
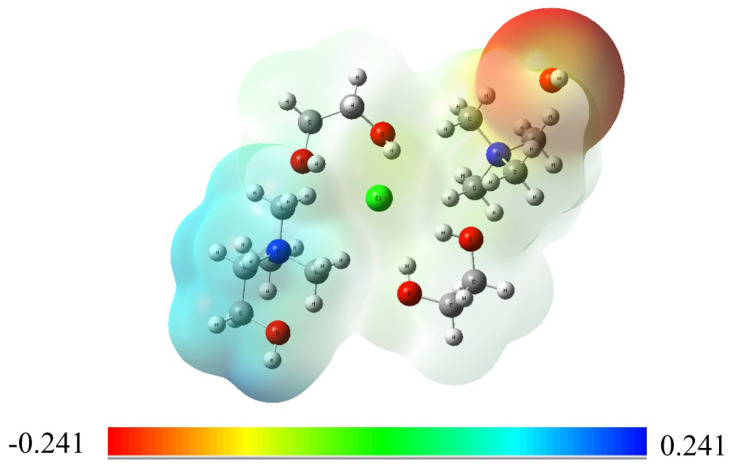
Molecular electrostatic potential map of the TMA head group in the presence of choline chloride and ethylene glycol at a 1:2 molar ratio, along with a ${\mathrm{OH}}^{-}$ ion. The map illustrates the charge distribution and interaction sites, showing the stabilizing effect of DES on the TMA head group by neutralizing its positive charge and reducing the reactivity of ${\mathrm{OH}}^{-}$ ions.

Molecular electrostatic potential (ESP) maps were employed to visualize the interaction between ${\mathrm{OH}}^{-}$ ions and the TMA head group in the presence of DES molecules, using the B3LYP DFT method. Initially, ${\mathrm{OH}}^{-}$ ions were positioned near the nitrogen atom of the TMA head group to neutralize its positive charge. The resulting charge distribution of the TMA head group, with the presence of DES, is displayed in Figure 2. The ESP map highlights the predominant interaction between ${\mathrm{OH}}^{-}$ ions and the nitrogen atom of the TMA head group, stabilizing the positive charge. This interaction is a critical factor in enhancing the chemical stability of the TMA head group in the DES environment.

Further analysis of the ESP map indicates that ${\mathrm{OH}}^{-}$ ions tend to occupy the space formed by the three methyl groups surrounding the nitrogen atom. This arrangement may contribute to a stabilizing effect of DES, as it could reduce the reactivity of ${\mathrm{OH}}^{-}$ ions toward the head group, potentially lowering the likelihood of nucleophilic attacks. In practical AEM applications, where the TMA head group is typically tethered to the polymer backbone, DES may offer a means to mitigate degradation under alkaline conditions. While these ESP maps offer useful insights into charge distribution and possible interaction sites, they represent a simplified view of the complex interactions at play. Further research will be required to fully understand the stabilizing effects of DES and its practical impact on AEM stability.

Under operational conditions in AEM fuel cells, increasing current density accelerates water consumption at the cathode, which can lead to a drier environment. In such conditions, ${\mathrm{OH}}^{-}$ ions that are not solvated by water molecules exhibit higher nucleophilicity, potentially accelerating the degradation of TMA head groups. Conversely, when ${\mathrm{OH}}^{-}$ ions are adequately solvated, their nucleophilicity is reduced, which underscores the importance of water content in influencing AEM stability. As water content decreases, the concentration of ${\mathrm{OH}}^{-}$ ions increases, potentially raising the risk of nucleophilic attacks on QA head groups. Understanding this degradation mechanism is important for evaluating the long-term performance of AEMs in fuel cell systems. Future studies that explore the effects of HLs and temperature will be valuable for refining our understanding of these interactions and their implications for AEM performance.

### 3.2. Activation and Reaction Energies

**Figure 3:**
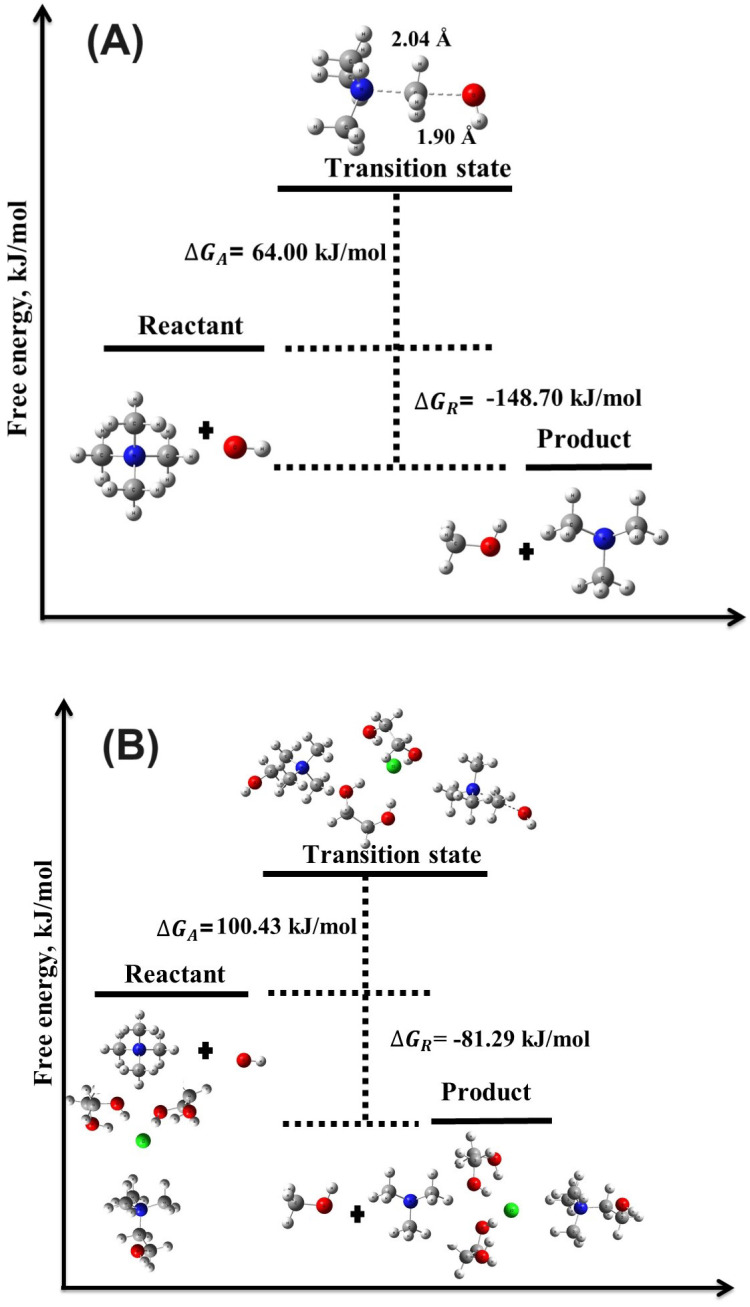
Illustration of the transition state structures and corresponding free energy barriers for the $S_{N}2$ mechanism of the TMA head group in the (**A**) absence and (**B**) presence of DES.

**Figure 4:**
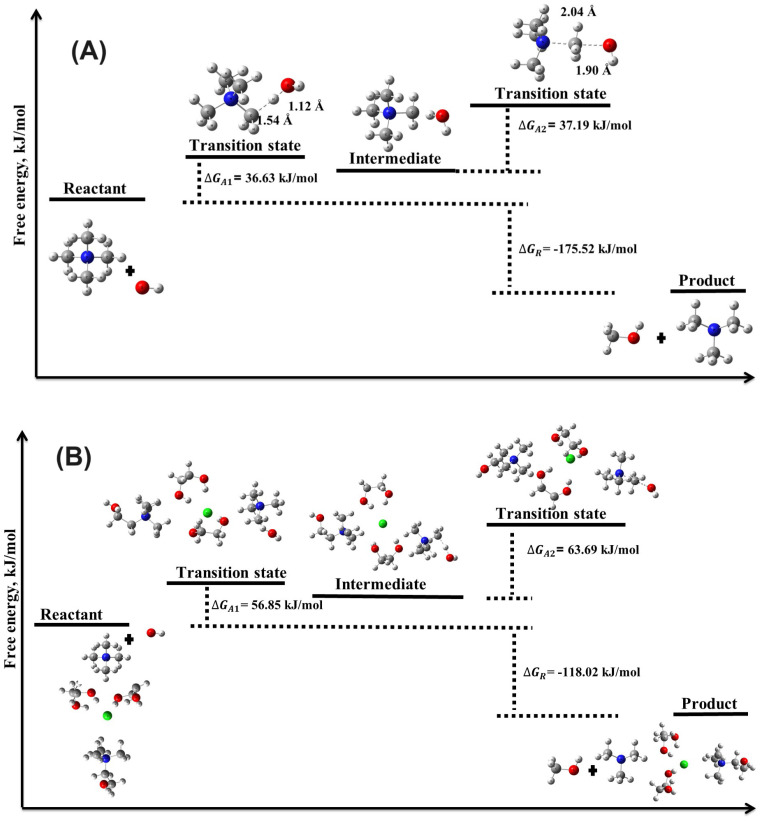
Illustration of the transition state structures and corresponding free energy barriers for the ylide formation mechanism of the TMA head group in the (**A**) absence and (**B**) presence of DES.

DFT calculations using the B3LYP hybrid functional were employed to investigate the chemical degradation reactions between ${\mathrm{OH}}^{-}$ ions and the TMA head group, focusing on the evaluation of reaction energies ($\Delta E_{\mathrm{reaction}}$, $\Delta G_{\mathrm{reaction}}$) and activation energies ($\Delta E_{\mathrm{activation}}$, $\Delta G_{\mathrm{activation}}$) for these reactions in both the absence and presence of DES. The results (Table 1, Figure 3 and Figure 4) provide crucial insights into the degradation mechanisms of TMA-based AEMs in alkaline environments, particularly highlighting the stabilizing role of DES. Compared to other theoretical studies, such as those by Chempath et al. [15,16], the calculated values of $\Delta G_{\mathrm{reaction}}$ and $\Delta E_{\mathrm{activation}}$ in the absence of DES align with previously reported data [15] for $S_{N}2$ and YF mechanisms. However, to the best of our knowledge, the role of DES in modifying these degradation mechanisms, specifically in terms of $\Delta E_{\mathrm{reaction}}$, $\Delta G_{\mathrm{reaction}}$, $\Delta E_{\mathrm{activation}}$, and $\Delta G_{\mathrm{activation}}$, has not been explored in the literature, marking this study’s contribution to understanding the role of DES in these reactions.

#### 3.2.1. Ylide Formation Mechanism

The YF degradation mechanism was analyzed in two distinct steps. In the first step, the activation energy barrier was found to be moderate, with $\Delta E_{\mathrm{activation}}$ of 18.02 kJ/mol and $\Delta G_{\mathrm{activation}}$ of 36.63 kJ/mol. The corresponding reaction energies ($\Delta E_{\mathrm{reaction}}$ and $\Delta G_{\mathrm{reaction}}$) were 16.95 kJ/mol and 26.81 kJ/mol, respectively, suggesting that while the reaction is thermodynamically favorable, it is less so compared to the $S_{N}2$ mechanism.

In the second step of the YF mechanism, we observed a significant decrease in both $\Delta E_{\mathrm{reaction}}$ and $\Delta G_{\mathrm{reaction}}$, with values of −135.21 kJ/mol and −175.52 kJ/mol, respectively. This indicates a highly exergonic reaction, favoring the spontaneous degradation of the TMA head group in the absence of DES. The activation energy barrier for this step was still relatively low, with $\Delta E_{\mathrm{activation}}=31.02$ kJ/mol and $\Delta G_{\mathrm{activation}}=37.19$ kJ/mol, further supporting the spontaneous nature of the YF mechanism.

#### 3.2.2. Nucleophilic Substitution Mechanism

The $S_{N}2$ mechanism displayed lower activation barriers than the YF mechanism, indicating it is more likely to occur under standard alkaline conditions. For the reaction without DES, the activation energy ($\Delta E_{\mathrm{activation}}$) was significantly lower than in the YF mechanism, with the reaction remaining spontaneous and exergonic. These findings align with previous research by Chempath et al. [15,16], which identified the $S_{N}2$ and YF mechanisms as key degradation pathways for TMA in the presence of ${\mathrm{OH}}^{-}$ ions. The low activation barriers in both mechanisms suggest that these pathways are prominent under alkaline conditions in the absence of DES.

#### 3.2.3. Impact of DES on Activation and Reaction Energies

In the presence of DES, a notable increase in the activation energy barriers was observed for both the YF and $S_{N}2$ mechanisms. For the $S_{N}2$ mechanism, $\Delta E_{\mathrm{activation}}$ increased to 54.62 kJ/mol, with a substantial rise in $\Delta G_{\mathrm{activation}}$ to 100.43 kJ/mol. Despite these higher energy barriers, the reaction remained exergonic and spontaneous, as reflected by $\Delta E_{\mathrm{reaction}}=-68.98$ kJ/mol and $\Delta G_{\mathrm{reaction}}=-81.29$ kJ/mol. This suggests that the presence of DES introduces a kinetic barrier that slows the degradation process, thereby improving the chemical stability of the TMA head group.

The effect of DES on the YF mechanism was similarly significant. In the first step of the YF mechanism, $\Delta E_{\mathrm{activation}}$ increased to 27.64 kJ/mol and $\Delta G_{\mathrm{activation}}$ to 56.85 kJ/mol, indicating that the presence of DES raises the energy barrier for this degradation pathway. Reaction energies also shifted towards more endergonic values, with $\Delta E_{\mathrm{reaction}}=27.66$ kJ/mol and $\Delta G_{\mathrm{reaction}}=36.73$ kJ/mol, suggesting that the DES environment stabilizes the system. In the second step of the YF mechanism, the presence of DES further increased both activation and reaction energies, with $\Delta E_{\mathrm{activation}}=39.97$ kJ/mol and $\Delta G_{\mathrm{activation}}=63.69$ kJ/mol. Although still exergonic, with $\Delta E_{\mathrm{reaction}}=-96.64$ kJ/mol and $\Delta G_{\mathrm{reaction}}=-118.02$ kJ/mol, the increased energy barriers highlight DES’s role in slowing the degradation process.

The increase in activation energy barriers in the presence of DES underscores its potential as a stabilizing agent for TMA head groups in AEMs. By altering the local ESP, DES raises the energy barriers for both $S_{N}2$ and YF degradation pathways, effectively mitigating the degradation process. This enhanced chemical stability is crucial for the long-term performance of AEMs in alkaline environments (Supplementary Materials).

The higher energy barriers observed in this study suggest that incorporating DES into AEM systems could significantly extend the operational lifetime of these membranes under alkaline conditions. To further explore the stability-enhancing role of DES, we conducted *ab initio* MD simulations to investigate the influence of HLs and temperature on degradation mechanisms. Understanding the impact of these conditions is essential for optimizing AEM performance in real-world operating environments, where moisture content and temperature fluctuations play a critical role in the chemical stability of QA head groups.

On the method side, although empirical dispersion corrections were not applied in the static DFT calculations, this decision was made to maintain consistency with previous studies that focused on similar QA head group systems without incorporating these corrections [13,15]. However, empirical dispersion corrections, such as Grimme D3, could provide additional accuracy by capturing long-range interactions, especially in systems where dispersion forces play a more pronounced role. This approach is employed in our *ab initio* MD simulations, where dispersion corrections accounted for intermolecular forces like hydrogen bonding and charge transfer in DES environments. While dispersion effects may be less significant for short-chain TMA cations, their inclusion in future studies, particularly for larger alkyl groups or different DES formulations, could further improve the accuracy of the thermodynamic and kinetic parameters. Studies such as Zaitsau et al. highlight the importance of accounting for dispersion forces in gas-phase calculations, especially for ionic systems [48].

### 3.3. Effect of Hydration Level

**Figure 5:**
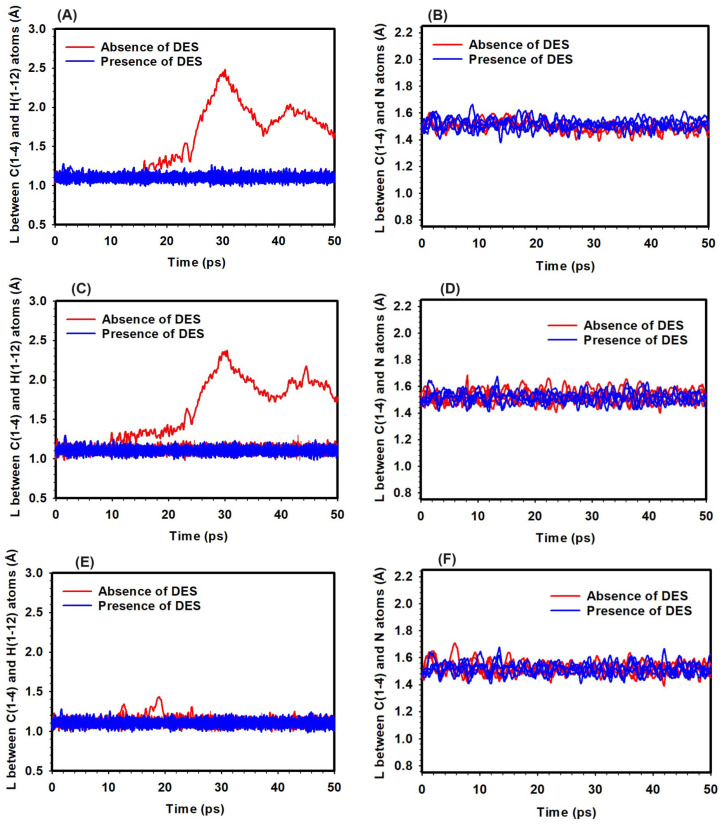
Bond distances in the TMA head group in the absence and presence of DES at HLs of 1 (**A**,**B**), 3 (**C**,**D**), and 5 (**E**,**F**). Left panels (**A**,**C**,**E**) show the bond distances between the H and C atoms, while right panels (**B**,**D**,**F**) depict the bond distances between the C and N atoms.

The influence of hydration level on the chemical degradation mechanisms of the TMA head group in the presence of ${\mathrm{OH}}^{-}$ ions was investigated. Bond-breaking events, particularly the C/N and H/C bond distances, were analyzed as indicators of potential $S_{N}2$ and YF reactions, leading to the production of methanol and trimethylammonium. The analysis covered three HLs: HL 1, HL 3 (representing typical operating conditions), and HL 5, both in the absence and presence of DES (Figure 5). To clarify, the TMA head group contains twelve C-H bonds and four C-N bonds, all of which are plotted in Figure 5 to show their bond distances over time under different hydration levels (HLs). Some of these bonds remained relatively stable and did not exhibit significant changes in bond distance during the simulation, leading to overlapping curves. However, for bonds where distance changes occurred, these variations are clearly visible and highlighted in the respective graphs.

At HL 1 and a temperature of 298 K, the C/N bond distance within the TMA head group remained stable at approximately 1.50 Å, indicating minimal bond-breaking events and low $S_{N}2$ reaction potential, regardless of DES presence (Figure 5). However, the H/C bond distance increased significantly from 1.10 Å to 2.12 Å in the absence of DES, suggesting potential YF activation. In contrast, the presence of DES maintained the H/C bond distance at around 1.10 Å, effectively suppressing the YF mechanism (Figure 5).

At HL 3 and 298 K, the C/N bond distance also remained stable, further supporting the low likelihood of $S_{N}2$ reactions under these conditions (Figure 5). The H/C bond distance, however, increased to 2.61 Å without DES, indicating a higher propensity for YF reactions. In the presence of DES, the H/C bond distance was stabilized at around 1.10 Å, inhibiting the YF mechanism (Figure 5).

At HL 5 and 298 K, the C/N bond distances remained stable, showing a low likelihood of $S_{N}2$ reactions at this hydration level (Figure 5). The H/C bond distance remained consistent around 1.10 Å, regardless of DES presence, indicating minimal YF mechanism activity at this hydration level and temperature (Figure 5).

The investigation provided insights into bond-breaking events within the TMA head group. Across all HLs at 298 K, minimal bond-breaking events were observed, indicating a low likelihood of $S_{N}2$ reactions. The YF mechanism, influenced by both HLs and the presence of DES, showed greater activation potential at lower HLs. However, DES effectively suppressed the YF mechanism by stabilizing the H/C bond distance. At higher HLs, the YF mechanism remained subdued regardless of DES, emphasizing the importance of hydration in controlling degradation pathways. These findings highlight the interplay between hydration and degradation mechanisms.

### 3.4. Effect of Temperature

**Figure 6:**
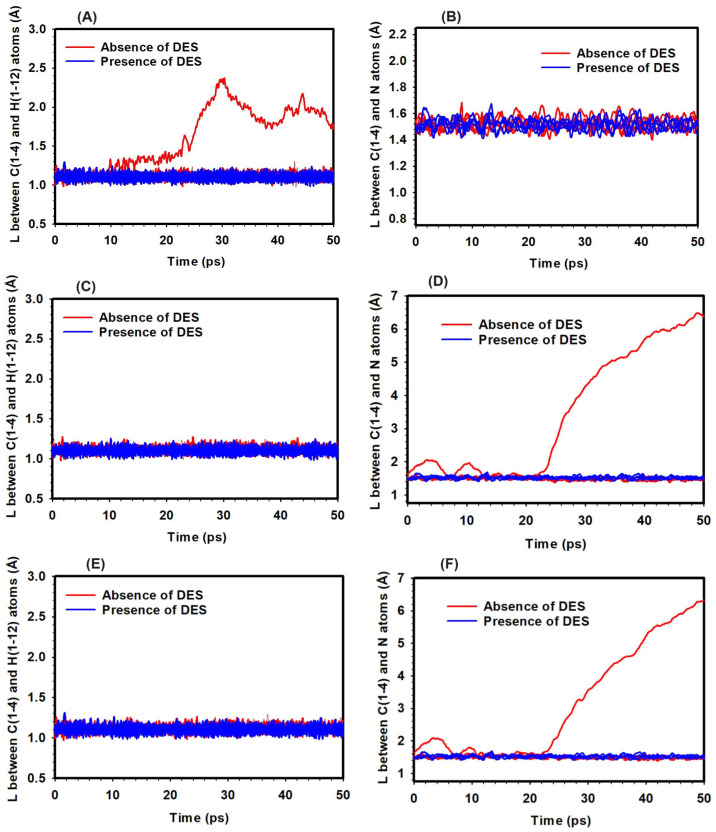
Bond distances in the TMA head group in the absence and presence of DES at HL 3 and varying temperatures: 298 K (**A**,**B**), 320 K (**C**,**D**), and 350 K (**E**,**F**). Left panels (**A**,**C**,**E**) show the bond distances between the H and C atoms, while right panels (**B**,**D**,**F**) depict the bond distances between the C and N atoms.

The effect of temperature on the bond distances within the TMA head group in the presence of ${\mathrm{OH}}^{-}$ ions was examined, with an emphasis on potential $S_{N}2$ and YF reactions. The study was carried out at three temperatures: 298 K, 320 K, and 350 K, both in the absence and presence of DES. The analysis tracked changes in the C/N and H/C bond distances (Figure 6). Since the TMA head group contains twelve C-H bonds and four C-N bonds, overlapping curves are observed for bonds that remained stable, while variations are clearly visible for bonds that showed significant changes during the simulation.

At 298 K and hydration level (HL) 3, the C/N bond distance remained at approximately 1.5 Å in both the presence and absence of DES, indicating that no significant bond-breaking events were observed that might suggest $S_{N}2$ reactions (Figure 6). In contrast, the H/C bond distance increased from 1.20 Å to 2.61 Å in the absence of DES, potentially indicating activation of the YF mechanism. With DES present, the H/C bond distance remained around 1.10 Å, suggesting that DES may have had a stabilizing effect (Figure 6).

At 320 K and HL 3, the C/N bond distance increased to 6.3 Å in the absence of DES, suggesting the occurrence of bond-breaking events and the potential for $S_{N}2$ reactions (Figure 6). In the presence of DES, the C/N bond distance remained stable at around 1.55 Å, with no significant bond-breaking observed. The H/C bond distance showed little variation, staying close to 1.10 Å in both cases, suggesting that YF activation was limited at this temperature (Figure 6).

At 350 K and HL 3, the C/N bond distance increased further to 6.56 Å without DES, indicating the possibility of bond-breaking events and $S_{N}2$ reactions (Figure 6). With DES present, the C/N bond distance stayed near 1.58 Å, and no significant bond-breaking was observed. Similar to previous results, the H/C bond distance remained consistent around 1.10 Å, regardless of DES, indicating minimal YF mechanism involvement at this temperature (Figure 6).

These observations suggest that temperature affects the likelihood of bond-breaking events in the TMA head group. The presence of DES appears to reduce the occurrence of these events across all temperatures studied, particularly in relation to $S_{N}2$ and YF reactions. While the YF mechanism may have been more active at lower temperatures, higher temperatures seemed to promote $S_{N}2$ reactions. These findings offer insight into the behavior of the TMA head group under varying thermal conditions, with the influence of DES observed to be a potential stabilizing factor.

## 4. Conclusions

In this study, we explored the chemical stability of TMA head groups, both with and without the presence of a choline chloride and ethylene glycol-based DES additives using DFT calculations and *ab initio* MD simulations. The investigation focused on key degradation mechanisms, such as $S_{N}2$ and YF, and the impact of DES addition on these mechanisms across different HLs and temperatures.

Our results indicate that DES effectively enhances the chemical stability of TMA head groups by consistently increasing the activation energy barriers for both $S_{N}2$ and YF mechanisms. In the absence of DES, YF was observed to dominate at lower HLs, while nucleophilic substitution became more prominent at elevated temperatures. However, in the presence of DES, both degradation mechanisms were significantly suppressed across all tested conditions, suggesting that DES acts as a stabilizing agent for the TMA head groups.

The ESP maps demonstrated that DES alters the interaction between ${\mathrm{OH}}^{-}$ ions and the TMA head group, reducing the reactivity of ${\mathrm{OH}}^{-}$ ions and thereby enhancing the chemical stability of the TMA groups. Additionally, DFT calculations showed that DES raised both reaction and activation energy barriers, further supporting the conclusion that DES plays a critical role in mitigating degradation.

Our *ab initio* MD simulations revealed that HLs strongly influence the stability of TMA head groups. YF was more likely at lower HLs in the absence of DES, while nucleophilic substitution became more probable at higher temperatures. The presence of DES, however, stabilized the TMA head groups under both conditions, reducing the likelihood of degradation.

Although DES additives seem to offer advantages in terms of enhancing the chemical stability of AEM head groups, their real-world application may still present challenges, particularly related to long-term stability and interactions with membrane components. Future experimental and computational studies should focus on optimizing DES composition to improve fluidity and compatibility with membrane materials, thereby enhancing their long-term stability and performance in practical applications.

In conclusion, the incorporation of DES into AEM systems offers the potential for improving the chemical stability of TMA head groups, particularly under alkaline conditions. Future work should focus on the experimental validation of these findings and the optimization of DES formulations for practical applications in AEMs, with the goal of enhancing membrane durability and performance in fuel cell technologies.

## Figures and Tables

**Table 1:** $\Delta E_{\mathrm{reaction}}$, $\Delta G_{\mathrm{reaction}}$, $\Delta E_{\mathrm{activation}}$, and $\Delta G_{\mathrm{activation}}$ values for the degradation reactions of TMA head group in the absence and presence of DES. All values are in the unit of kJ/mol.

	$S_{N}2$		YF (Step 1)		YF (Step 2)	
	**Without DES**	**With DES**	**Without DES**	**With DES**	**Without DES**	**With DES**
$\Delta E_{\mathrm{reaction}}$	−105.47	−68.98	16.95	27.66	−135.21	−96.64
$\Delta E_{\mathrm{activation}}$	48.18	54.62	18.02	27.64	31.02	39.97
$\Delta G_{\mathrm{reaction}}$	−148.70	−81.29	26.81	36.73	−175.52	−118.02
$\Delta G_{\mathrm{activation}}$	64.00	100.43	36.63	56.85	37.19	63.69

## Data Availability

The data and materials are available from the authors upon request.

## Supplementary Materials

The following supporting information can be downloaded at: https://www.mdpi.com/article/10.3390/molecules29204869/s1, The Supplementary Material includes additional information on simulation settings. extended DFT calculation results, geometries of reactants at maximum separation distances, transition state and final state geometries from static DFT calculations, and a detailed description of the designed AIMD systems. Table S1 provides the number of choline chloride and ethylene glycol molecules used in the AIMD simulations, along with other relevant system parameters. The supplementary material also includes energy and free energy tables (Tables S2–S5) for DES-supported systems, as well as graphical depictions of the relevant geometries (Figures S1–S4). This supplementary information supports and enhances the findings discussed in the main manuscript.

## Abbreviations

The following abbreviations are used in this manuscript:

AEM	Anion Exchange Membrane
BSSE	Basis Set Superposition Error
DES	Deep Eutectic Solvent
DFT	Density Functional Theory
$\Delta E_{\mathrm{reaction}}$	Reaction Energy
$\Delta E_{\mathrm{activation}}$	Activation Energy
$\Delta G_{\mathrm{reaction}}$	Reaction Free Energy
$\Delta G_{\mathrm{activation}}$	Activation Free Energy
HL	Hydration Level
MD	Molecular Dynamics
${\mathrm{OH}}^{-}$	Hydroxide Ion
QA	Quaternary Ammonium
YF	Ylide Formation
$S_{N}2$	Nucleophilic Substitution
