# Supplementary material for: Enhanced Chemical Stability of Tetramethylammonium Head Groups via Deep Eutectic Solvent: A Computational Study
Source: Molecules. 2024 Oct 14;29(20):4869. doi: 10.3390/molecules29204869 (PMC11509940; doi:10.3390/molecules29204869)
Supplement: Supplementary file 1 [file molecules-29-04869-s001.zip › molecules-3232913-supplementary.pdf]

Supporting Materials for "Atomistic modeling of tetramethylammonium head groups supported by Deep Eutectic Solvents for Anion Exchange Membranes"

Mirat Karibayev[a], Bauyrzhan Myrzakhmetov[b], Yanwei Wang[a,b,*], Almagul Mentbayeva[a,*]

[a] *Department of Chemical and Materials Engineering, School of Engineering and Digital Sciences, Nazarbayev University, 53 Kabanbay Batyr Avenue, Astana, 010000, Kazakhstan*

[b] *Center for Energy and Advanced Materials Science, National Laboratory Astana, 53 Kabanbay Batyr Avenue, Astana, 010000, Kazakhstan*

S1. Additional simulation settings

Ab initio MD simulation setup was created by placing one tetramethylammonium (TMA) head group and one negatively charged OH^- ion, along with 1-5 water molecules at 298-350 K, in the absence and presence of choline chloride (ChCl) and ethylene glycol (EGL) based DES as shown in Table S1.

Namely, Table S1 presents a comprehensive overview of our designed systems, each characterized by operating temperatures ranging from 298 K to 350 K. The systems include TMA head group in both pure forms and when supported by Deep Eutectic Solvents (DES). The table outlines the composition of each system in terms of the presence of an head groups, OH^- ion, water molecules, choline chloride (ChCl), ethylene glycol (EGL), and the total number of atoms in the simulation.

Distinct configurations are presented for varying water content, demonstrating the influence of the hydration on the system. Furthermore, the inclusion of ChCl and EGL in DES-supported systems indicates the role of these components in altering the system's characteristics. The temperature parameter allows for a thorough examination of the systems' behavior under

*Corresponding author

Email address: yanwei.wang@nu.edu.kz (Y.W); almagul.mentbayeva@nu.edu.kz (A.M) (Almagul Mentbayeva)

Table S1: Description for our designed systems at 1.50 × 1.50 × 1.50 nm^3, 298-350 K.

	AEM	OH^-	Water	ChCl	EGL	Number of atoms	T (K)
TMA	1	1	1	-	-	22	298
DES supported TMA	1	1	1	1	2	64	298
TMA	1	1	3	-	-	28	298
DES supported TMA	1	1	3	1	2	70	298
TMA	1	1	3	-	-	28	320
DES supported TMA	1	1	3	1	2	70	320
TMA	1	1	3	-	-	28	350
DES supported TMA	1	1	3	1	2	70	350
TMA	1	1	5	-	-	34	298
DES supported TMA	1	1	5	1	2	76	298

different thermal conditions. This table serves as a valuable reference for understanding the diverse compositions and conditions of the designed systems, offering insights into their structural variations and potential applications in a range of environments.

S2. Supplementary Results from DFT calculations

Tables S2 and S3 provide energy values for our designed systems with DES supported AEM, as determined through DFT calculations. The table presents various energy components, including reaction energies (E(R1) and E(R2)), transition states, BSSE, and product energies (E(P1) and E(P2). The negative and positive signs indicate the absence and presence of DES, respectively.

Table S2: Energy values for our designed systemsof YF degradation for DES supported AEM studied by the DFT calculations.

	E(R1)	E(R2)	E(TS(1))	E(Inter(P1))	E(Inter(P2))	E(TS(2))	E(P1)	E(P2)
(-) DES	-562666.04	-199429.14	-762064.38	-561301.01	-200764.45	-762036.01	-458238.23	-303962.42
(+) DES	-3844567.10	-199429.14	-4043955.57	-3843204.12	-200764.45	-4043930.35	-3740102.80	-303962.42

Table S3: Energy values for our designed systems of S_N2 degradation for DES supported AEM studied by the DFT calculations.

	E(R1)	E(R2)	E(TS1)	BSSE	E(P1)	E(P2)
(-) DES	-562666.04	-199429.14	-762036.01	11.00	-458238.23	-303962.42
(+) DES	-3844567.10	-199429.14	-4043930.35	11.27	-3740102.80	-303962.42

Tables S4 and S5 present the free energy values for the systems we designed with DES-supported AEM, as obtained from DFT frequency calculations. These tables detail several free energy components, including the reaction free energies, transition states, and product free energies. The signs (negative and positive) denote the absence or presence of DES, respectively.

Table S4: Free energy values for our designed systems of YF degradation reaction for DES supported AEM studied by the DFT calculations.

	G(R1)	G(R2)	G(TS(1))	G(inter(P1))	G(inter(P(2)))	G(TS2)	G(P1)	G(P2)
(-) DES	-562286.91	-199448.95	-761699.23	-560952.15	-200756.89	-761671.85	-457996.09	-303888.47
(+) DES	-3843096.68	-199448.95	-4042428.79	-3841752.01	-200756.89	-4042445.21	-3738738.45	-303888.47

Table S5: Free energy values for our designed systems of S_N2 degradation for DES supported AEM studied by the DFT calculations.

	G(R1)	G(R2)	G(TS)	G(P1)	G(P2)
(-) DES	-562286.91	-199448.95	-761671.85	-457996.09	-303888.47
(+) DES	-3843096.68	-199448.95	-4042445.21	-3738738.45	-303888.47

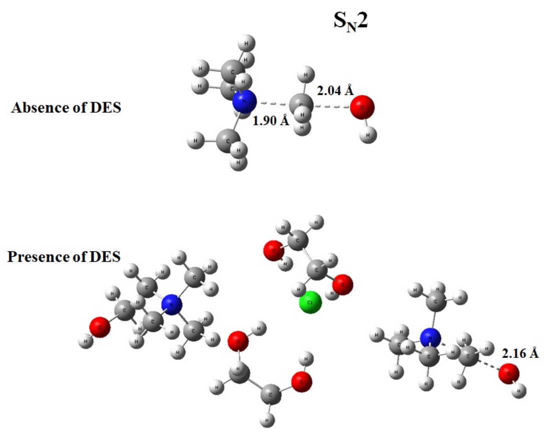

Figure S1: Depiction of the relevant geometries for TMA head group segments in the absence and presence of DES from DFT calculations (S_N2).

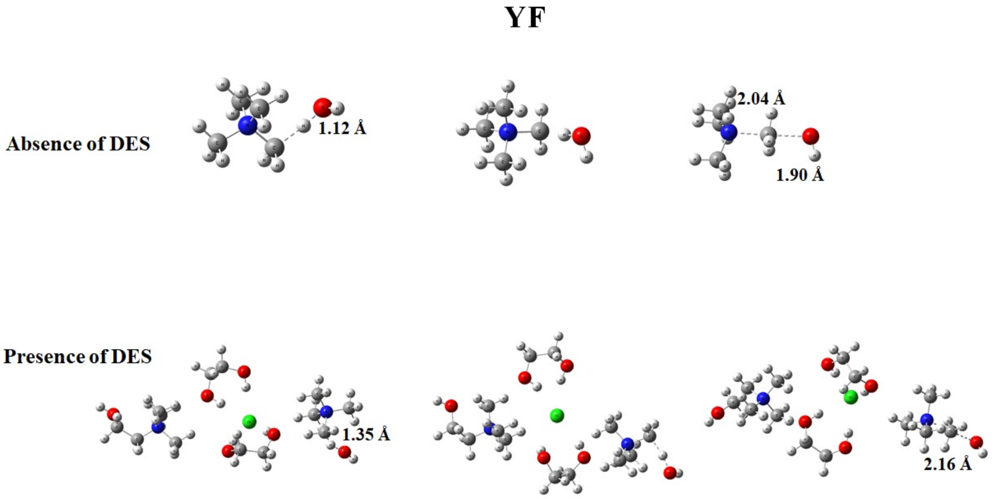

Figure S2: Depiction of the relevant geometries for TMA head group segments in the absence and presence of DES from DFT calculations (YF).

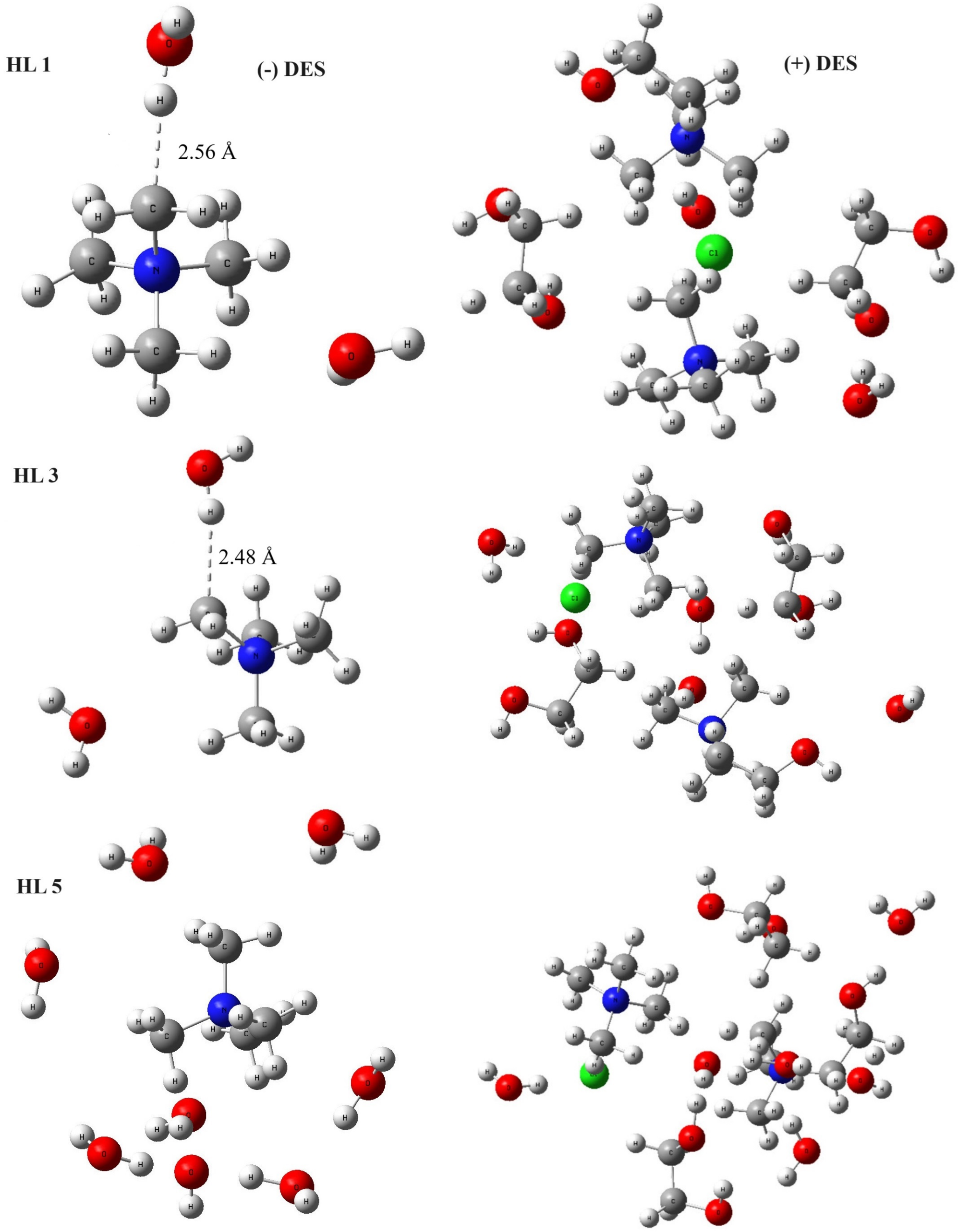

Figure S3: Depiction of the relevant geometries for TMA head group segments in the absence and presence of DES from AIMD work at the different HLs.

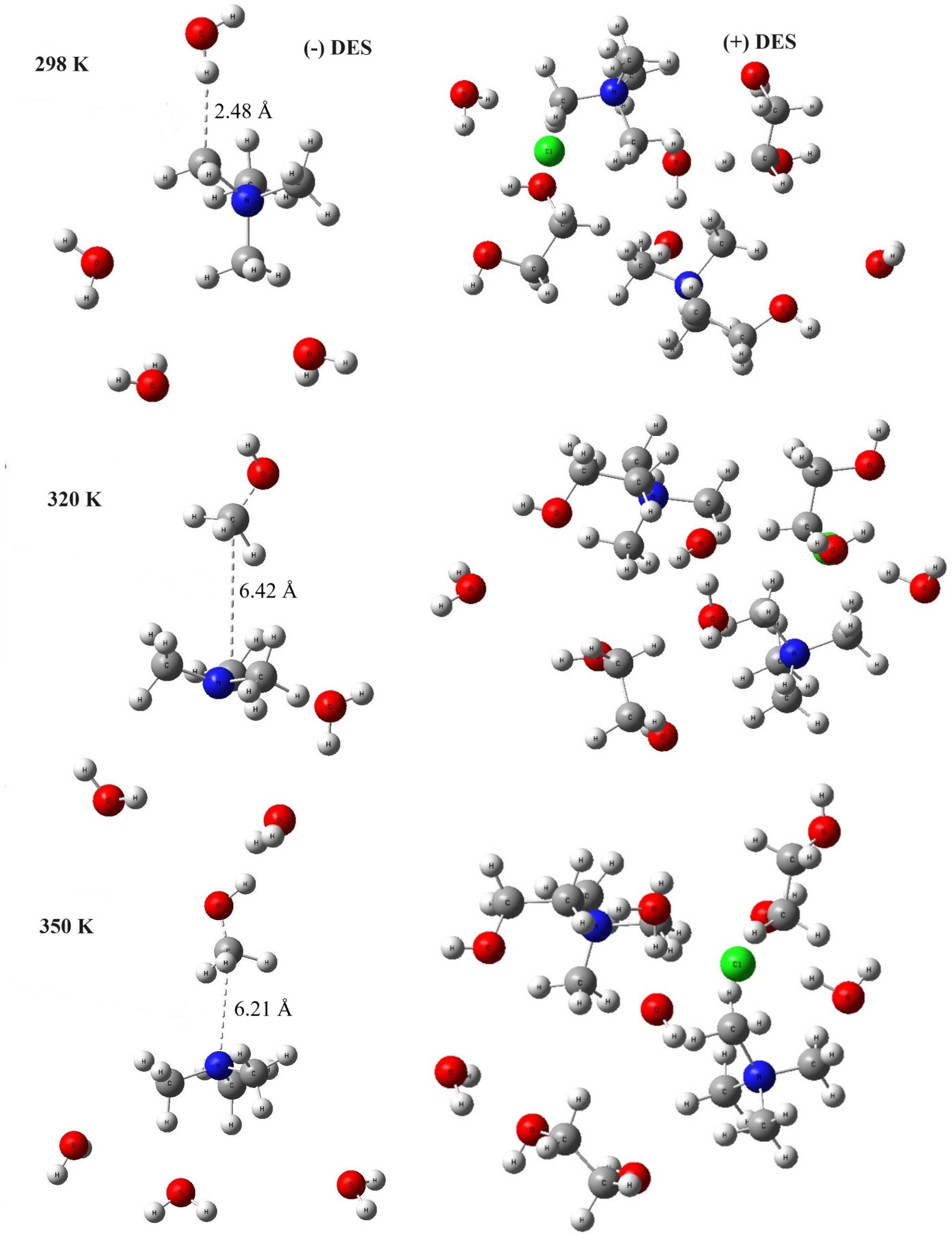

Figure S4: Depiction of the relevant geometries for TMA head group segments in the absence and presence of DES from AIMD work at the different temperatures.
